# 靶向CD19 CAR-T细胞序贯阿扎胞苷联合维奈克拉治疗混合表型急性白血病异基因造血干细胞移植后复发1例

**DOI:** 10.3760/cma.j.cn121090-20241203-00528

**Published:** 2025-05

**Authors:** 晓嘉 郭, 漾 杨, 高翔 王, 阳 曹, 晓霞 胡, 传和 江

**Affiliations:** 1 兰州大学第二医院血液科，兰州 730030 Department of Hematology, Lanzhou University Second Hospital, Lanzhou 730030, China; 2 上海交通大学医学院附属瑞金医院血液科，上海血液学研究所，国家转化医学中心，上海 200025 State Key Laboratory of Medical Genomics, Shanghai Institute of Hematology, National Research Center for Translational Medicine, Shanghai Rui Jin Hospital, Shanghai Jiao Tong University School of Medicine, Shanghai 200025, China; 3 华中科技大学同济医学院血液科，武汉 430030 Department of Hematology, Tongji Hospital, Tongji Medical College, Huazhong University of Science and Technology, Wuhan 430030, China

患者，女性，34岁，因“头晕”于2022年11月18日就诊，诊断为混合表型急性白血病（MPAL）伴KMT2A::AFF1融合基因阳性。诱导化疗（环磷酰胺+长春地辛+甲泼尼龙+伊达比星+阿糖胞苷）1个疗程获得完全缓解（CR），流式细胞术微小残留病（MRD）阴性。甲氨蝶呤+阿糖胞苷方案巩固化疗2个疗程。移植前疾病评估CR，MRD 0.14％（B系），髓系MRD阴性，KMT2A::AFF1融合基因阳性，中枢神经系统白血病阴性。2023年5月接受单倍体造血干细胞移植，供者为表兄，HLA 6/12相合，血型B+供B+，供者特异性抗体（DSA）阴性。预处理方案为依托泊苷+氟达拉滨+白消安+美法仑，移植物抗宿主病（GVHD）预防方案为抗人胸腺细胞球蛋白+环孢素A+霉酚酸酯+甲氨蝶呤，CD34^+^细胞输注量8.3×10^6^/kg，单个核细胞输注量4.7×10^8^/kg。移植后13 d粒系植入，17 d血小板植入。

移植后1、2个月原发病评估均为CR，流式细胞术MRD及KMT2A::AFF1融合基因均阴性。移植后3个月（8月4日）骨髓CR，流式细胞术分析示异常幼稚B淋巴细胞0.32％，KMT2A::AFF1融合基因0.704％，完全供者嵌合，停环孢素A，予以干扰素抢先治疗。2周后复查骨髓原始细胞44.5％，流式细胞术分析示异常B系34.93％（表达CD19、CD58、CD22、HLA-DR），未见髓系异常细胞，KMT2A::AFF1融合基因54.731％。诊断血液学复发，加用奥加伊妥珠单抗继续治疗。9月14日再次复查：骨髓原幼细胞11.5％，流式细胞术分析示异常B系15.56％。2023年9月21日外周血流式细胞术分析示异常B淋巴细胞6.6％，表达CD19、HLA-DR、CD38^dim^、CD22^dim^、cCD79a、cTdT、CD123^dim^。评估治疗无效，外周血T细胞嵌合95.4％，拟进行经靶向CD19嵌合抗原受体T（CAR-T）细胞挽救治疗。10月5日起予以氟达拉滨+环磷酰胺方案预处理，回输患者自身来源CD19 CAR-T细胞1.5×10^6^/kg，回输后10 d外周血CAR-T细胞扩增达峰，42 d骨髓流式细胞术分析B系和髓系MRD均阴性，KMT2A::AFF1融合基因阴性。治疗过程中发生2级细胞因子释放综合征（CRS），未发生GVHD。CAR-T细胞回输后45 d起予以阿扎胞苷联合维奈克拉方案维持治疗4个疗程。持续监测患者的MRD和CAR-T动力学，患者CAR-T细胞存续7个月，随访至CD19 CAR-T细胞治疗后1年，原发病评估CR，流式细胞术MRD及KMT2A::AFF1融合基因定量阴性，无GVHD表现。

讨论：MPAL属于相对少见的白血病类型，目前尚无标准治疗方案。欧洲骨髓移植登记处一项纳入500例成人MPAL的大宗回顾性分析显示，即使接受allo-HSCT，MPAL患者3年总生存率也仅为56.3％，复发率高达31.4％。除特殊亚型外（如伴有BCR::ABL1融合基因），MPAL移植后尚缺乏有效复发预防手段。本例患者为移植后B系单系复发，接受靶向CD19 CAR-T细胞治疗再次获得B系CR，序贯应用对B系、髓系均有作用的阿扎胞苷联合维奈克拉方案，获得了较好的无事件生存。

